# Cell-Specific Transcriptional Responses to Heat Shock in the Mouse Utricle Epithelium

**DOI:** 10.3389/fncel.2020.00123

**Published:** 2020-05-15

**Authors:** Erica Sadler, Matthew M. Ryals, Lindsey A. May, Daniel Martin, Nora Welsh, Erich T. Boger, Robert J. Morell, Ronna Hertzano, Lisa L. Cunningham

**Affiliations:** ^1^Section on Sensory Cell Biology, National Institute on Deafness and Other Communication Disorders (NIDCD), National Institutes of Health, Bethesda, MD, United States; ^2^Department of Molecular Biology and Genetics, The Johns Hopkins University School of Medicine, Baltimore, MD, United States; ^3^Genomics and Computational Biology Core, National Institute on Deafness and Other Communication Disorders (NIDCD), National Institutes of Health, Bethesda, MD, United States; ^4^Genomics and Computational Biology Core, National Institute of Dental and Craniofacial Research, National Institutes of Health, Bethesda, MD, United States; ^5^Department of Otorhinolaryngology-Head and Neck Surgery, University of Maryland School of Medicine, Baltimore, MD, United States; ^6^Department of Anatomy and Neurobiology, University of Maryland School of Medicine, Baltimore, MD, United States; ^7^Institute for Genome Sciences, University of Maryland School of Medicine, Baltimore, MD, United States

**Keywords:** heat shock, Ribotag, RNA-seq, supporting cell, hair cell, utricle

## Abstract

Sensory epithelia of the inner ear contain mechanosensory hair cells (HCs) and glia-like supporting cells (SCs), both of which are required for hearing and balance functions. Each of these cell types has unique responses to ototoxic and cytoprotective stimuli. Non-lethal heat stress in the mammalian utricle induces heat shock proteins (HSPs) and protects against ototoxic drug-induced hair cell death. Induction of HSPs in the utricle demonstrates cell-type specificity at the protein level, with HSP70 induction occurring primarily in SCs, while HSP32 (also known as heme oxygenase 1, HMOX1) is induced primarily in resident macrophages. Neither of these HSPs are robustly induced in HCs, suggesting that HCs may have little capacity for induction of stress-induced protective responses. To determine the transcriptional responses to heat shock of these different cell types, we performed cell-type-specific transcriptional profiling using the RiboTag method, which allows for immunoprecipitation (IP) of actively translating mRNAs from specific cell types. RNA-Seq differential gene expression analyses demonstrated that the RiboTag method identified known cell type-specific markers as well as new markers for HCs and SCs. Gene expression differences suggest that HCs and SCs exhibit differential transcriptional heat shock responses. The chaperonin family member *Cct8* was significantly enriched only in heat-shocked HCs, while *Hspa1l* (HSP70 family), and *Hspb1* and *Cryab* (HSP27 and HSP20 families, respectively) were enriched only in SCs. Together our data indicate that HCs exhibit a limited but unique heat shock response, and SCs exhibit a broader and more robust transcriptional response to protective heat stress.

## Introduction

The inner ear contains six highly specialized sensory epithelia that are responsible for auditory and vestibular functions. Each epithelium contains mechanosensory hair cells (HCs) that transduce sound energy or head movement into neural input to the brain. Surrounding the HCs in each sensory epithelium are glia-like cells collectively referred to as supporting cells (SCs). SCs serve a variety of functions, including functional and structural support, clearance of extracellular debris and dying HCs, and formation of scars that seal the reticular lamina after hair cell death (Forge, 1985; Abrashkin et al., 2006; Bird et al., 2010; Anttonen et al., 2014; Monzack et al., 2015). SCs also perform other glial functions including providing trophic support to neurons (Montcouquiol et al., 1998; Sugawara et al., 2007) and clearing neurotransmitter from the synaptic cleft between HCs and primary afferent neurons (Glowatzki et al., 2006). Together these functions of SCs promote hair cell mechanoelectrical transduction and synaptic function. In both the auditory and vestibular systems, the stromal tissue beneath the sensory epithelium contains resident tissue macrophages as well as bone marrow-derived macrophages identified by macrophage markers (CX3CR1, IBA1; Okano et al., 2008; Sato et al., 2010). These cells migrate to the sensory epithelium to assist SCs in phagocytizing dead and dying HCs and cellular debris following hair cell death (Kaur et al., 2015; Hirose et al., 2017).

HCs, SCs, and resident macrophages show differential responses to stresses (Francis and Cunningham, 2017). For example, HCs are more susceptible than SCs to damaging stimuli, including ototoxic drugs and noise damage. Similarly, these cell types demonstrate differential responses to protective stimulation, including non-lethal heat shock. We showed previously that heat shock induces heat shock proteins (HSPs) in the mouse utricle *ex vivo*, and this HSP induction protects against ototoxic drug-induced hair cell death (Cunningham and Brandon, 2006; Taleb et al., 2008). HSP70 expression is both necessary and sufficient for this protective effect (Taleb et al., 2009; Baker et al., 2015). In response to heat shock, we observe robust induction of HSP70 immunoreactivity in SCs with little induction in HCs (May et al., 2013; Breglio et al., 2020). Similarly, pharmacological induction of heme oxygenase-1 (HMOX1, also called Heat Shock Protein 32, HSP32) protects against both aminoglycoside- and cisplatin-induced hair cell death (Francis et al., 2011; Baker et al., 2015), but HMOX1 immunoreactivity is observed in resident macrophages in the underlying stroma of the utricle, not in HCs or SCs (Baker et al., 2015). Thus, HSP-mediated protection is non-cell-autonomous in the inner ear (May et al., 2013; Francis and Cunningham, 2017). To better understand the full range of cell-type-specific responses to protective heat stress and to determine whether these differential responses are transcriptionally regulated, we performed cell-type-specific transcriptional profiling of these cell types in response to heat shock.

Several methods of generating cell-type-specific transcriptional profiles have been used in the inner ear, including FACS-sorting of dissociated cells (Hertzano et al., 2011; Tao and Segil, 2015; Waldhaus et al., 2015; Hickox et al., 2017; Lush et al., 2019), manual collection of individual cells by pipette (Liu et al., 2014; Ranum et al., 2019), and single-cell capture (Burns et al., 2015; Durruthy-Durruthy and Heller, 2015; Durruthy-Durruthy et al., 2018; Yamashita et al., 2018; Korrapati et al., 2019; Yu et al., 2019) followed by RNA-Seq or mass spectrometry and translatome analysis utilizing the RiboTag system (Sanz et al., 2009; Chessum et al., 2018; Matern et al., 2018). The first two approaches require dissociation of the sensory epithelium, which can alter gene expression and induce stress response genes, including HSPs (van den Brink et al., 2017). Conversely, the RiboTag approach utilizes a transgenic mouse bearing a floxed wild-type exon 4 followed by an alternate exon 4 with a hemagglutinin (HA) tag in the *Rpl22* ribosomal protein locus. When crossed to a transgenic mouse expressing a Cre-driver in the cell types of interest, the wild-type exon is excised, and the HA-tagged exon is brought in frame in the resulting transcript. This method allows isolation of cell-specific transcripts *via* immunoprecipitation (IP) of the HA-tagged ribosomal subunit RPL22 directly from lysed tissue, without requiring dissociation and cell isolation, thereby avoiding the cellular stress caused by dissociation. Characterization of the RNA isolated from the IP thus reveals a subset of the transcripts actively being translated from the cell types of interest at the time of capture, i.e., a sample of that cell’s “translatome.” This technique was previously used to study the transcriptomes of other difficult-to-isolate cell types such as Sertoli cells in the mouse testis and HCs in zebrafish, and was shown to avoid the induction of immediate early genes (De Gendt et al., 2014; Matern et al., 2018).

Two Cre lines were selected for this study: Gfi1-Cre and GLAST-CreER. Growth Factor Independent 1 Transcriptional Repressor (GFI1) is involved in HC development and survival (Hertzano et al., 2004), and Gfi1-Cre (Yang et al., 2010) is expressed in HCs and macrophages in the inner ear (Matern et al., 2017). Gfi1-Cre has been used to drive fluorescent protein expression in HCs, to isolate neonatal utricle HCs for single-cell RNA-Seq analysis (Burns et al., 2015), and to drive expression of genetic markers of HC development (Liu et al., 2012). Special consideration of the Cre line used to isolate utricle SCs was necessary, because SCs share a common progenitor with HCs (Lanford et al., 1999), and SCs retain a limited ability to transdifferentiate into HCs (White et al., 2006; Lin et al., 2011; Sinkkonen et al., 2011; Bramhall et al., 2014; Franco and Malgrange, 2017; McGovern et al., 2019), especially in the utricle (Wang et al., 2015; Bucks et al., 2017). Therefore, we used an inducible Cre model for SCs to allow for Cre induction in mature SCs. Sodium-Dependent Glutamate/Aspartate Transporter 1 (GLAST, aka SLC1A3) is a glutamate transporter expressed in juvenile and adult SCs (Jin et al., 2003; Glowatzki et al., 2006; Dalet et al., 2012). The GLAST-CreER mouse bears a tamoxifen-inducible Cre transgene (Wang et al., 2012), and this model has been used to induce recombination in SCs of the cochlea (Mellado Lagarde et al., 2014). We crossed the RiboTag mouse with Gfi1-Cre mice in order to obtain HC-specific transcripts, and with GLAST-CreER mice to obtain SC-specific transcripts. RiboTag immunoprecipitated transcripts were isolated from control and heat shocked utricles, and the transcriptional responses of each cell type to heat shock were characterized by RNA-Seq.

## Materials and Methods

### Mouse Breeding, Organotypic Utricle Culture, Heat Shock Stimulation

Gfi1-Cre [Gfi1tm1(cre)Gan] mice were generated by Dr. Lin Gan at U. Rochester, and they were generously provided for this study by Dr. Matthew W. Kelley, Laboratory of Cochlear Development, National Institute on Deafness and Other Communication Disorders. GLAST-CreER mice [Tg(Slc1a3-cre/ERT)1Nat; Stock #012586], RiboTag mice (B6N.129-Rpl22tm1.1Psam/J; Stock # 011029), and CBA/J mice (Stock # 000656) were obtained from the Jackson Laboratory. Male Gfi1-Cre, GLAST-CreER, and RiboTag mice were each bred with female wild-type CBA/J mice for a single generation. Genotyping was performed using genotyping primers previously described (Yang et al., 2010) or the primers suggested by the Jackson Laboratory. Mice that were positive for at least one copy of either Gfi1-Cre or GLAST-CreER were then crossed to mice with at least one copy of the RiboTag Rpl22-HA. Mice from the second cross were genotyped again, and experiments for hair cell-specific transcripts were performed with utricles from mice bearing both Gfi1-Cre and Rpl22-HA. Experiments for supporting cell-specific transcripts were performed using utricles from mice bearing both GLAST-CreER and Rpl22-HA. All mice used in this study were adults, with ages ranging from P30-P60. A mixture of male and female mice was used in all experiments. Ribotag IP experiments used 8–10 whole utricles (or 10–12 peeled epithelia) in each biological replicate with exactly half from males and half from females.

Mice were euthanized by CO_2_ asphyxiation followed by decapitation. Utricles were immediately dissected in M199 media (Life Technologies, 12350039), and the epithelial roof was gently dissected away, leaving the otoconia intact. The utricles were incubated overnight at 37°C (95% air/5% CO_2_) in DMEM/F-12 media (Life Technologies, Carlsbad, CA, USA, 11320033) with 5% FBS (Thermo Fisher Scientific, Waltham, MA, USA), and 50 U/ml penicillin (Sigma–Aldrich, St. Louis, MO, USA). Cultured utricles were then either exposed to heat shock in microcentrifuge tubes placed in a 43°C water bath for 30 min and allowed to recover for 2 h, or they remained at 37°C under control culture conditions. Some utricles were further dissected to remove the macrophage-containing stromal tissue underlying the sensory epithelium. For this procedure, cultured utricles were incubated with thermolysin (1–2 mg/ml; Sigma), elastase (4 U/ml; Sigma), and DNase I (10 Kunitz/ml; Ambion, Austin, TX, USA) in serum-free DMEM/F12 media for 10–15 min at 37°C. Following treatment, utricles were transferred into a petri dish containing serum-free DMEM-F12, and epithelia were carefully isolated (“peeled”) from the underlying stroma using an eyelash tool. Stroma-free isolated sensory epithelia were then flash-frozen. All animal procedures were approved by the NIH/NINDS Animal Care and Use Committee.

### *GLAST-CreER* Tamoxifen Induction and *Cre* Line Reporter Characterization

To confirm the cell type-specificity of the Cre lines, each line of Cre mice was initially crossed to a reporter line, B6.Cg-Gt(ROSA)26Sortm14(CAG-tdTomato)Hze/J (hereafter referred to as Rosa26-tdTomato, obtained from the Jackson Laboratory, stock #007914). Following genotyping of pups, mice positive for both GLAST-CreER and Rosa26-tdTomato were administered either tamoxifen (30 mg/ml in corn oil; Sigma, T5648) at 0.225 mg/g body weight or an equivalent volume of corn oil vehicle by IP injection at P21–22 as previously described (Mellado Lagarde et al., 2014). Utricles were analyzed 3 weeks post-injection. Utricles from mice bearing both Rosa26-tdTomato and either Gfi1-Cre or GLAST-CreER were used to quantify Cre expression. Following overnight fixation (4% PFA in 1× PBS (Thermo Fisher Scientific) and three 15-min washes in 1× PBS), tdTomato fluorescence in utricle whole mounts was used to quantify the percentage of tdTomato-positive cells in each Cre reporter cross following staining for Myosin-VIIa and Hoechst 33342 (see “Immunohistochemistry” section for immunohistochemistry methods). Z-stack images (1 μm step size, unidirectional scanning, two frame averaging) were obtained for each utricle by imaging through the sensory epithelium using a Zeiss LSM 780 confocal microscope (Carl Zeiss Microscopy, Oberkochen Germany). Image analysis was performed using Zen 2.3 software (Carl Zeiss Microscopy). tdTomato-positive HCs were counted in five 2,500 μm^2^ regions, and the percentage of those cells displaying Myosin-VIIa immunoreactivity at the level of the hair cell body and nucleus were averaged across regions and reported as hair cell density. tdTomato-positive SCs were counted at the level of the supporting cell nuclei in five regions, and the percentage of those cells out of the total number of nuclei in the region were averaged across regions and reported as supporting cell density. While no marker was used to differentiate striolar vs. extrastriolar regions of the utricle, all supporting cell images were taken from the periphery of the utricle, which is presumed to be extrastriolar. The number of tdTomato-positive HCs and tdTomato-positive SCs in Gfi1-Cre mice were compared using an unpaired *t*-test. The number of tdTomato-positive HCs and SCs in GLAST-CreER mice with and without tamoxifen injection were compared using an unpaired *t*-test with Welch’s correction due to a significant difference in variances.

### Immunohistochemistry

For validation studies, IHC was performed using utricles from adult (P30–60) wild-type CBA/J mice (both males and females). Utricles were stained and mounted either as whole mounts or as 10 μm frozen sections. For whole mount utricle experiments, Alexa Fluor-647 Phalloidin (1:75; Thermo Fisher Scientific, A22287) was used to label the cuticular plate and stereocilia. All whole mount utricles undergoing IHC were fixed overnight at 4°C or for 1 h at room temperature with 4% PFA in 1× PBS (Thermo Fisher Scientific), washed three times (3×), 15 min each, with 1× PBS at room temperature followed by incubation in blocking solution (1× PBS, 2% bovine serum albumin, 0.8% normal goat serum or normal donkey serum, and 0.4% Triton X-100) for 3 h at room temperature. All sectioned utricles were fixed with 4% PFA in 1× PBS (Thermo Fisher Scientific) for 1 h prior to freezing and sectioning. Prior to staining, sectioned samples were fixed for an additional 5 min in the same fixing solution, washed 3×, 10 min each, with 1× PBS at room temperature followed by incubation in the same blocking solution for 3 h at room temperature. All utricles were then incubated in primary antibody overnight at 4°C, washed 3× with blocking solution, and incubated in secondary antibody for 4 h at RT. Whole mounted utricles were then counterstained for 10 min with Hoechst 33342 (1:5,000–20,000; Thermo Fisher Scientific, H3570), washed 3× for 15 min 1× PBS, and mounted on glass slides using Fluoromount G (Southern Biotech, Birmingham Alabama). Sectioned utricles were mounted using ProLong Glass Antifade Mountant with NucBlue Stain (Thermo Fisher Scientific, P36981). Imaging was performed using a Zeiss LSM 780 confocal microscope (Carl Zeiss Microscopy). The following primary antibodies were used for IHC and visualized using AlexaFluor-conjugated secondary antibodies (1:500; Thermo Fisher Scientific): mouse anti-Myo7a (1:100, Developmental Studies Hybridoma Bank, Iowa City Iowa, 138-1), rabbit anti-Myo7a (1:250; Proteus Biosciences, Ramona, CA, USA 25-6790), rabbit anti-Rbp1 (1:100; Abcam, Cambridge, UK, ab154881), mouse anti-Tspan8 (1:100; Thermo Fisher Scientific, MA5-24296), goat anti-Rbm24 (1:100; Santa Cruz Biotech, Dallas Texas, SC-248361), rabbit anti-Calb1 (1:100; Thermo Fisher Scientific, 711443), rabbit anti-Cct8 (1:400; Abcam, ab96321), and rabbit anti-HSP27 (1:500; Millipore Sigma, Darmstadt Germany, 06-517).

### Ribotag Immunoprecipitation, cDNA Library Preparation, RNA Sequencing, Alignment of Reads, and Bioinformatic Workflow

**Figure 1:**
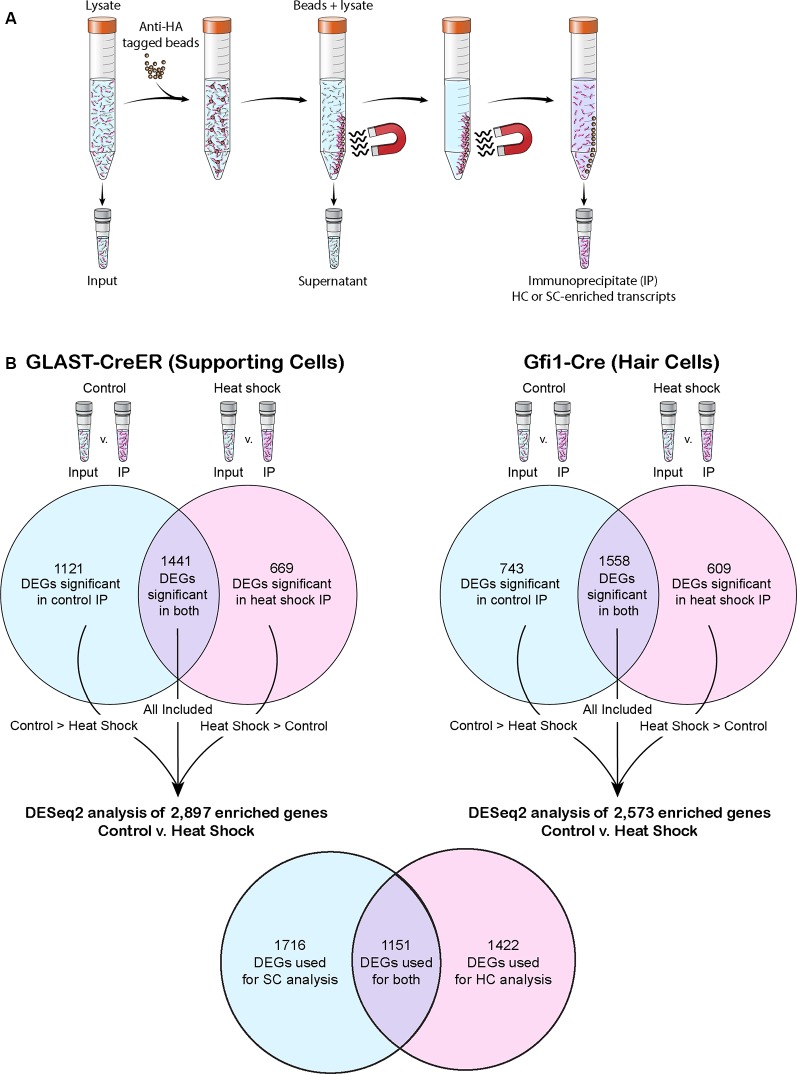
The RiboTag precipitation method and post-RiboTag gene comparison performed in this study. **(A)** The RiboTag precipitation method uses anti-hemagglutinin (HA) tagged beads to select for HA-tagged ribosomes. Because only ribosomes of the cell type selected for by the Cre driver are HA-tagged, the resulting immunoprecipitate (IP) contains cell type-specific transcripts. **(B)** Transcripts of each experiment’s IP were compared to transcripts of the corresponding input in order to computationally remove any background (non-cell specific transcripts) not successfully removed as part of the supernatant. When comparing across conditions or drivers, only genes found to be significant in the IP of both conditions or significant in the IP of the condition in which that transcript had greater mean expression were included in the analysis. Venn diagrams depict the number of differentially expressed genes (DEGs) that reached significance in one or both conditions of control and heat shock for each Cre driver. The total number of genes used in each control vs. heat shock DESeq2 analysis is listed, and the number of genes these analyses had in common is shown in the final Venn diagram.

For IP, 8–10 pooled whole utricles (or 10–12 peeled epithelia) were used in each biological replicate. Immunoprecipitation was performed on four biological replicates of GLAST-CreER utricles in both control and heat shock conditions, three biological replicates of whole tissue Gfi1-Cre utricles in both control and heat shock conditions, and two biological replicates of the stroma-free isolated epithelium of Gfi1-Cre utricles. Ribosome IP was performed as described previously (Sanz et al., 2009; Chessum et al., 2018) with a minor modification. Briefly, the utricles were flash-frozen, homogenized in a Dounce homogenizer, and then incubated for 6 h at 4°C with a mouse anti-HA monoclonal antibody (Covance, MMS-101R). Antibody-incubated lysate was then precipitated using Protein G Dynabeads (Invitrogen, Carlsbad, CA, USA) and incubated at 4°C overnight. RNA was extracted from the initial lysate (hereafter referred to as “input”), the IP samples, and the remaining lysate (hereafter referred to as “supernatant”) using the RNeasy Micro Plus kit (Qiagen, Hilden Germany) including the genomic DNA removal spin column step (Figure 1A). RNA concentration and integrity of each IP and input RNA sample were determined using a total RNA Pico chip on a Bioanalyzer (Agilent Technologies, Santa Clara, CA, USA). Sequencing libraries were prepared using the SMART-seq v4 Ultra Low Input RNA Kit for Sequencing (Takara Bio USA, Mountain View, CA, USA). Dual indexed libraries were prepared using the Nextera XT DNA Library Preparation kit (Illumina, San Diego, CA, USA). Eighteen Gfi1-Cre samples, including input, IP, and supernatants, were multiplexed and sequenced on a HiSeq 1500 (Illumina) in 126 × 126 bp paired end mode. On a second flow cell, 24 GLAST-CreER and stroma-free isolated (“peeled”) sensory epithelium Gfi1-Cre samples (input and IP only for each condition) were multiplexed along with a repeat of the original 18 Gfi1-Cre samples and run on a HiSeq 1500 (Illumina) using 126 × 126 bp paired end mode.

### Differential Gene Expression Analysis Workflow and Gene Ontology Analysis

Demultiplexed FASTQ files were mapped to the mouse GRCm38/mm10 genome (Gencode GRCm38.vM11) using the STAR (v2.5.2) aligner (Dobin et al., 2013) with the “GeneCounts” parameter. Raw gene counts (Supplementary Table S3) were analyzed for differential gene expression using the RNA-Seq statistical method DESeq2 (Love et al., 2014). One control GLAST-CreER IP out of four replicates was excluded from further analysis because it was an outlier in almost every technical aspect, having the smallest library, lowest alignment rate, and highest 3’ bias. For each experiment, genes were first tested for significant enrichment in the IP vs. the matching input sample, where significance was defined as a Bonferroni-corrected *p*-value of 0.05 or less and enrichment of at least 2-fold (Supplementary Table S4). When analyzing the data within each cell-type enrichment experiment (i.e., from either the GLAST-Cre or Gfi1-Cre crosses), only those genes that were identified as significantly enriched in the IP over input, in the condition (either control or heat shock) that exhibits larger mean expression of that gene, were considered for downstream analyses (Figure 1B). To identify DEGs, significance was again defined as a Bonferroni-corrected *p*-value of 0.05 or less and expression change of at least 2-fold. Relevant comparisons such as control isolated-epithelium Gfi1-Cre IP vs. control whole-tissue GLAST-CreER IP were used to analyze cell-type-specific marker expression and validate cell type specificity based on Cre driver (Supplementary Table S5). All data used for DE analyses are available in GEO (GSE139593) and gEAR^1^ databases. For PCA analysis and visualization, the PCAExplorer package version 2.10.1 was used (Marini and Binder, 2019). For gene-ontology (GO) annotation analysis, the PANTHER Classification System (Mi et al., 2013, 2017) was used in conjunction with the GO Ontology database (released on 2018-12-03). GO annotations for the input genes were assigned a Bonferroni-corrected *p-value* and fold-enrichment compared to GO annotations in the *Mus musculus* PANTHER database of 22,262 mouse genes using Fisher’s Exact test. The “Complete Molecular Function” GO ontology database was used to determine which GO annotations were overrepresented in each gene set using a cut off of a Bonferroni-corrected *p*-value of 0.05 or less and a 2-fold enrichment cut off. For differential GO annotation analyses between IP groups, ToppCluster (Kaimal et al., 2010) was used in conjunction with the GO ontology database. REViGO (Supek et al., 2011) was used to remove redundant Gene Ontology terms when greater than 10 GO annotations were found significant. Adjusted *p*-values were included, and the SimRel score (Schlicker et al., 2006) was used as the semantic similarity measure.

## Results

### Crosses With Reporter Mice Reveal Epithelial Hair Cell-Specific Recombination Using Gfi1-Cre and Supporting Cell-Specific Recombination Using GLAST-CreER

**Figure 2:**
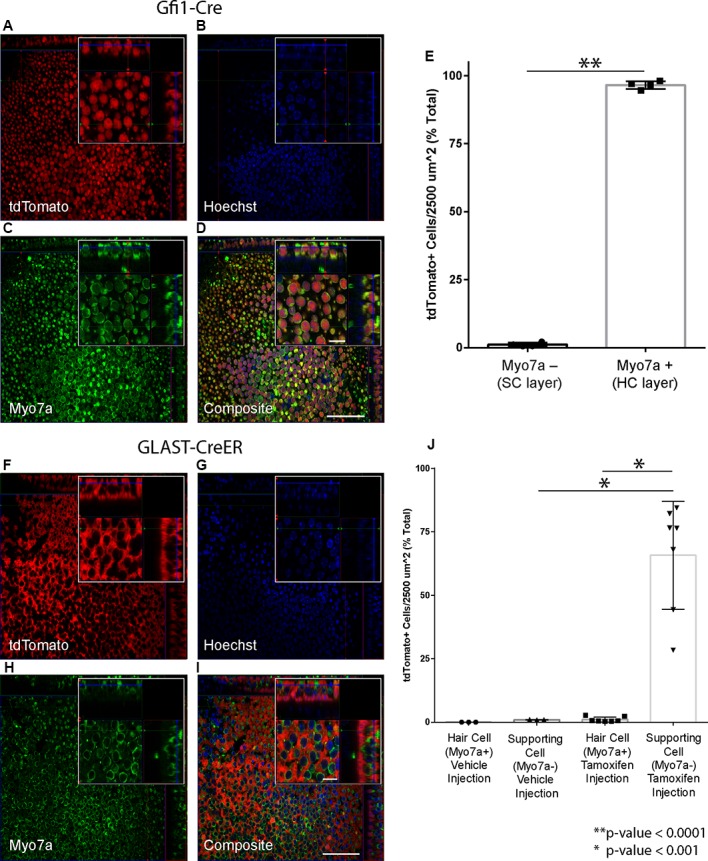
Hair cell (HC) and supporting cell (SC)-specific recombination using the Cre drivers selected for the study. **(A–E)** Gfi1-Cre results in recombination in HCs. **(A–D)** Representative maximum intensity projections from a Gfi1-Cre x Rosa26-tdTomato mouse utricle showing tdTomato expression **(A)**. Myo7a **(C)** was used as a hair cell marker to count HCs, and Hoechst staining **(B)** was used to count SC nuclei. Composite image **(D)** shows localization of the tdTomato signal primarily in HCs. **(E)** 96.5% of HCs and 1.1% of SCs are tdTomato+ in utricles from Gfi1-Cre;Rosa26-tdTomato mice. **(F–I)** GLAST-CreER results in recombination in SCs. Representative maximum intensity projections from a tamoxifen-injected GLAST-CreER;Rosa26-tdTomato mouse showing tdTomato expression **(F)**, Myo7a (hair cell marker) staining **(H)**, Hoechst **(G)**, and a composite **(I)** immunostaining. Localization in SCs is observed in the composite image. **(J)** Quantification of tdTomato expression in cells in both vehicle-injected and tamoxifen-injected mice showing that tamoxifen results in tdTomato induction in SCs with little induction in HCs. Scale bars **(I)** represent 50 μm (large panel) and 10 μm (small panel) and apply to all panels of the same respective size.

The selected Cre lines were crossed with reporter mice to confirm cell-type-specific recombination in the utricle. Utricles from Gfi1-Cre;Rosa26-tdTomato mice showed robust tdTomato labeling in nearly all HCs of the epithelial layer of the sensory epithelium (Figures 2A–D), with 96.5% (SD ± 1.5%; *n* = 4) of HCs expressing tdTomato (Figure 2E). Utricles from these mice showed very little tdTomato-positive signal in the SC layer, with an average density of 1.1% (SD ± 0.7%; *n* = 4) tdTomato-positive SCs per 2,500 μm^2^ (Figure 2D). These data indicate that Gfi1-Cre resulted in robust recombination in HCs with very little recombination in SCs.

Utricles from GLAST-CreER;Rosa26-tdTomato mice that were injected with corn oil vehicle showed no tdTomato-positive HCs (*n* = 3), and an average density of 1.0% (SD ± 0.1%; *n* = 3) tdTomato-positive cells in the SC layer. Utricles from mice that received tamoxifen at P21–22 (Figures 2F–I) showed expression of tdTomato in the SC layer, with an average of 65.8% (SD ± 21.3%; *n* = 7) of SCs expressing tdTomato (Figure 2J). Induction of tdTomato in HCs of these mice was low at 1.1% (SD ± 1.0%; *n* = 7; Figure 2J). These data indicate that while the GLAST-CreER did not result in recombination in all SCs, recombination was specific to SCs with very little induction in HCs. There were no obvious regional differences in recombination throughout the utricle. This aligns well with previous studies showing that GLAST-CreER has limited efficiency in SCs but results in little to no recombination in HCs and no significant difference in recombination between the peripheral vs. central regions of the utricle (Stone et al., 2018). Additionally, immunogold labeling for GLAST is present in SCs in contact with both type I and type II vestibular HCs in adult rats, confirming GLAST is expressed in all types of SCs in the utricle at adult age (Takumi et al., 1997).

### Transcriptomes of RiboTag IPs Readily Separate According to Both Cell Type and Experimental Condition

**Figure 3:**
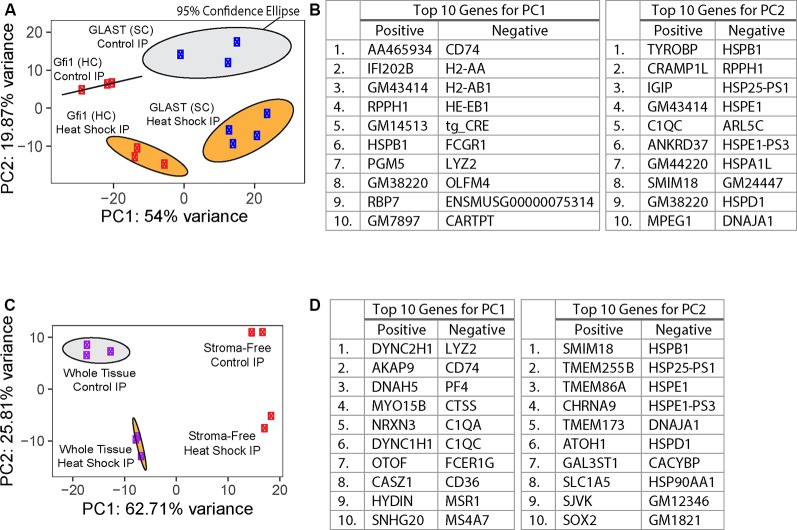
Principal component analysis (PCA) of RiboTag IP samples. **(A)** PCA of whole-tissue IP samples from RiboTag Gfi1-Cre (red) and GLAST-CreER (blue) IPs. PC1 represents 54% of the total variance in the experimental data, and PC2 represents 19.87% of the total variance. Ellipses represent 95% confidence ellipses around each group of samples, and the color of each ellipse corresponds to the treatment type (heat shock in orange or control in gray). **(B)** Tables show the 10 genes with the highest loadings in either direction for the two PCs plotted in **(A)**. **(C)** PCA of IP samples from RiboTag Gfi1-Cre whole-tissue (purple) and stroma-free (red) IPs. PC1 represents 62.71% of the total variance in the experimental data, and PC2 represents 25.81% of the total variance. Ellipses represent 95% confidence ellipses around each group of at least three samples, and the color of each ellipse corresponds to the treatment type (heat shock in orange or control in gray). **(D)** Tables show the 10 genes with the highest loadings in either direction for the two PCs plotted in **(C)**.

HC- and SC-specific RNA-Seq was performed using total RNA isolated from cultured utricles; cDNA libraries were generated and sequenced as described in “Materials and Methods” section. Using the top 500 most variable genes, principal component analysis (PCA) of the control whole tissue Gfi1-Cre IP (*n* = 3 biological replicates consisting of 8–12 utricles per replicate), control whole tissue GLAST-CreER IP (*n* = 3), heat shock whole tissue Gfi1-Cre IP (*n* = 3), and heat shock whole tissue GLAST-CreER IP (*n* = 4) separated groups based on the Cre driver along principal component 1 (PC1; 54% of the total variance) and treatment type (heat shock or control) along principal component 2 (PC2; 19.87% of the total variance; Figure 3A). The top 10 genes driving PC1 toward Gfi1-Cre IP samples (the negative direction) included immune-related genes *Cd74*, *H2-Aa*, *H2-Ab1*, *H2-Eb1*, *Fcgr1*, *Lyz2*, and *Olfm4* as well as *Cartpt* which is expressed in HCs (Scheffer et al., 2015), suggesting a strong presence of macrophages in addition to HCs in the whole-tissue Gfi1-Cre IP samples. *tg_CRE* was also a top 10 driver toward Gfi1-Cre IP samples along PC1. However, it should be noted that both the Gfi1-Cre transgene and the GLAST-CreER transgene mapped to the same Cre cassette in the reference genome used in this study. The top 10 genes driving PC2 toward heat-shocked IP samples (the negative direction) included genes associated with known HSPs *Hspb1*, *Hspe1*, *Hspa1l*, *Hspd1*, and *Dnaja1* (Figure 3B). The 95% confidence ellipses plotted within the PC1 and PC2 coordinate space show a clear separation of each experimental IP group. Examination of additional PCs revealed that 73.87% of the cumulative variance was captured within the first two PCs. Based on this separation, we analyzed DEGs with confidence that each group represented a unique combination of variance in treatment and cell type.

### Removing the Underlying Stroma From the Sensory Epithelium Reveals a Macrophage DEG Signature in the Gfi1-Cre RiboTag IP

**Figure 4:**
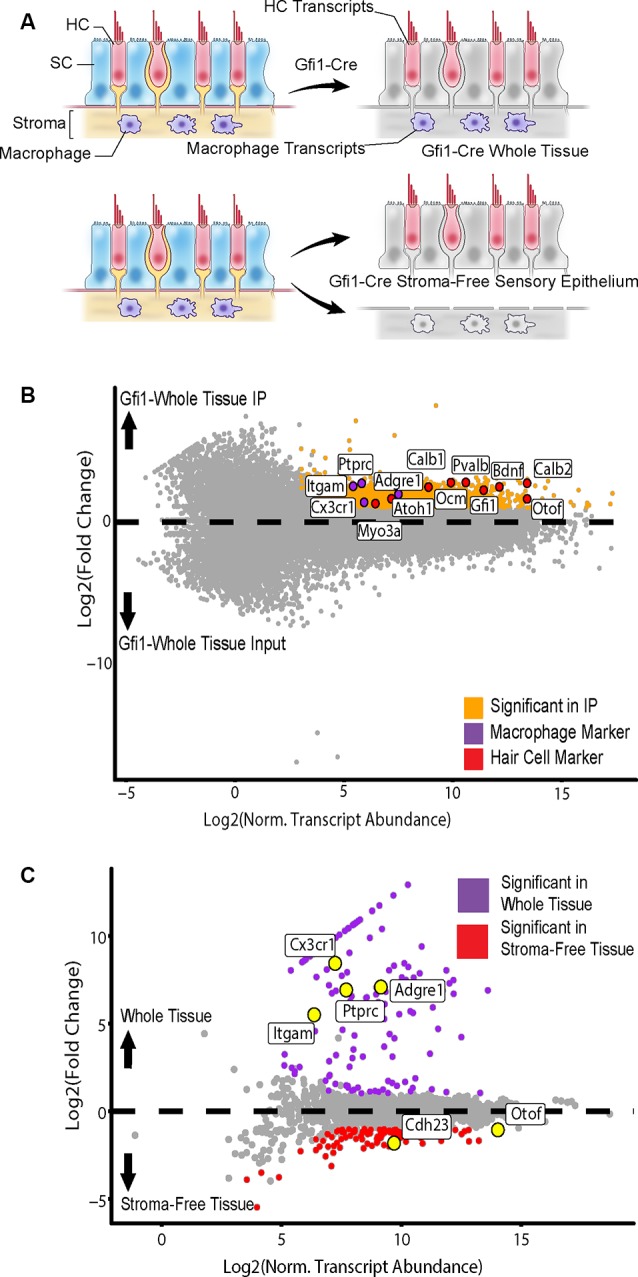
Isolation of cell type-specific transcripts from both HCs and tissue macrophages in the Gfi1-Cre RiboTag model. **(A)** Identification of macrophage-specific transcripts was achieved by comparing differentially expressed genes (DEGs) from whole utricle Gfi1-Cre RiboTag IPs (top) to those from utricles in which the stroma (containing resident macrophages) had been removed from the sensory epithelium, yielding a stroma-free isolated sensory epithelium (bottom). Shown are transcripts from HCs (red), SCs (blue), stroma (beige), and macrophages (purple). **(B)** Comparison of whole utricle Gfi1-Cre IP to input DEGs revealed enrichment for both hair cell and macrophage markers. Scatterplot shows Log_2_FC values vs. normalized transcript abundance from DEG comparison of the whole tissue Gfi1-Cre IP to the input. Markers of HC type (*Myo3a, Atoh1, Calb1, Ocm, Pvalb, Gfi1, Calb2, Bdnf, Otof*) are shown in red and labeled along with markers of tissue macrophage cell type shown in purple [*Itgam* (CD11b), *Ptprc* (CD45), *Cx3cr1*, *Adgre1* (F4/80)]. **(C)** Comparison of whole tissue (sensory epithelium plus stroma, purple) to isolated sensory epithelium (without stroma, red)DEGs revealed macrophage markers. Scatterplot shows Log_2_FC values vs. normalized transcript abundance from DEG comparison of the whole tissue Gfi1-Cre IP to the isolated sensory epithelium Gfi1-Cre. Markers of HC type (*Otof*, *Cdh23*) are labeled along with markers of tissue macrophage cell type [*Itgam* (CD11b), *Ptprc* (CD45), *Cx3cr1*, *Adgre1* (F4/80)].

In addition to HCs, Gfi1-Cre induces Cre recombination in resident macrophages of the inner ear, including those in the stromal tissue beneath the utricle sensory epithelium (Matern et al., 2017). To determine if transcripts from macrophages were present in the IPs from Gfi1-Cre RiboTag mice, we compared transcripts isolated from Gfi1-Cre IPs (*n* = 3 biological replicates consisting of 8–12 utricles per replicate) to those isolated from utricles in which we removed the stromal tissue (containing the resident macrophages) and examined transcripts from the remaining stroma-free isolated sensory epithelium (*n* = 2; Figure 4A). PCA analysis using the top 500 most variable genes revealed that principal component 1 (PC1; 62.71% of the total variance) separates samples based on whether the samples were whole utricle (including stroma) or isolated sensory epithelium (Figure 3C). The top 10 genes driving PC1 toward whole-tissue IP samples (the negative direction) were all genes known to be expressed in macrophages: *Lyz2*, *Cd74*, *Pf4*, *Ctss*, *C1qa*, *C1qc*, *Fcer1g*, *Cd36*, *Msr1*, and *Ms4a7*. The top 10 genes driving PC1 toward stroma-free, isolated sensory epithelium IPs included hair cell markers *Dync2h1*, *Dnah5*, *Nrxn3*, *Otof*, and *Casz1* (Figure 3D). Thus, the primary difference in DEGs in a comparison between these two groups reveals transcript differences between whole tissue (with stroma) and isolated sensory epithelium (without stroma). PCA separated groups based on treatment type (heat shock or control) along principal component 2 (PC2; 14.84% of the total variance). The top 10 genes driving PC2 toward heat shock included the known HSPs *Hspb1*, *Hspe1*, *Dnaja1*, *Hspd1*, and *Hsp90aa1*.

The whole tissue IP showed enrichment for both hair cell and macrophage markers in comparison to the input (Figure 4B). 142 DEGs were enriched in the whole tissue compared to the stroma-free isolated epithelia, and 117 DEGs were enriched in the isolated epithelia compared to the whole tissue (Figure 4C). The group of 142 DEGs enriched in the whole tissue group contained 21 genes enriched in the GO annotation “inflammatory response” (ToppCluster GO annotation, FDR < 0.05, GO:0006954) and 14 genes with the GO annotation “immune system development” (FDR < 0.05, GO:0002520). These included *Ptprc* (also known as CD45), a general immune cell marker, and markers of tissue macrophage identity including *Cx3cr1*, *Itgam* (also known as CD11b), and *Adgre1* (also known as F4/80; Okano et al., 2008; Sato et al., 2010; O’Malley et al., 2016; Matern et al., 2017; Figure 4C). Importantly, neither of these inflammation-related GO annotations was enriched in the stroma-free isolated epithelia DEG set, which was enriched for “ear development” (FDR < 0.05, GO:0043583). Thus, isolating the sensory epithelium from the underlying stromal tissue allowed us to identify macrophage-specific transcripts in the Gfi1-Cre RiboTag IP. In all subsequent analyses, only stroma-free isolated epithelium samples were used with the Gfi1-Cre in order to examine hair cell transcriptional responses without contamination from macrophages.

### Transcripts From the Gfi1-Cre IP Include Canonical Markers of Hair Cells, and Transcripts From the GLAST-CreER IP Include Canonical Markers of Supporting Cells

**Figure 5:**
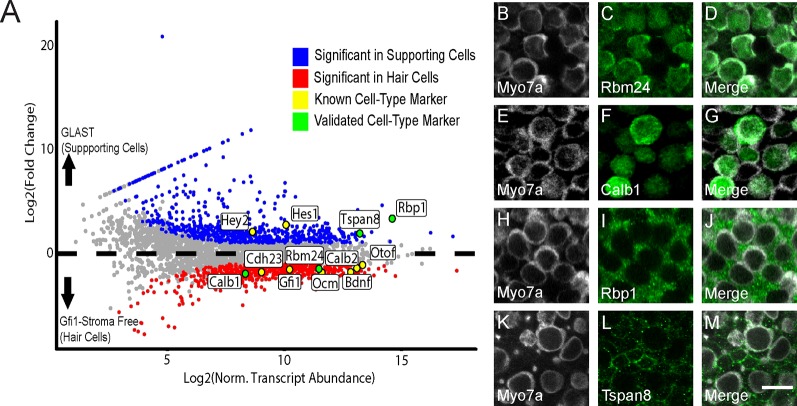
Identification of previously-known and newly-validated cell-type-specific transcripts. **(A)** Gfi1-Cre RiboTag IPs are enriched for canonical HC markers, and GLAST-CreER RiboTag IPs are enriched for SC markers. Scatterplot of Log_2_FC values vs. normalized transcript abundance from the comparison of the Gfi1-Cre IP to the GLAST-CreER IP in the control (no heat shock) condition. Some known HC and SC markers are labeled (yellow) as well as transcripts selected for validation (green). **(B–L)** Immunohistochemical staining for the four targets selected in **(A)**. *Rbm24* (green, **C**) and *Calb1* (green, **F**) staining is observed in HCs. *Myo7a* (white) was used as a known HC marker **(B,E)** with merged images **(D,G)**. *Rbp1* (green, **I**) and *Tspan8* (green, **L**) are observed in SCs.* Myo7a* (white) was used as a known HC marker **(H,K)** with merged images **(J,M)**. Images are 900 μm^2^ composites from confocal images taken at 63× magnification. Scale bar **(M)** represents 10 μm and applies to all panels.

Figure 5 shows the DEGs that were enriched in the Gfi1-Cre IP (isolated epithelium) compared to the GLAST-CreER IP under control (no heat shock) conditions. 685 DEGs were enriched in the hair cell-specific IP. These enriched transcripts included well-known HC markers such as *Gfi1*, *Ocm*, *Calb1*, *Calb2*, *Bdnf*, *Cdh23*, and *Otof* (Figure 5A). *Calb2* is preferentially expressed in Type II HCs (Desai et al., 2005) while *Ocm* is preferentially expressed in Type I HCs (Simmons et al., 2010), suggesting the transcripts of both HC subtypes were enriched. These data indicate that the Gfi1-Cre RiboTag IP is enriched for HC-specific transcripts. 729 DEGs were enriched in the control GLAST-CreER IP. Known markers of SCs, including *Hey2* and *Hes1* were enriched in the GLAST-CreER IP (Figure 5A). Thus, the GLAST-CreER RiboTag IP is enriched for supporting cell-specific transcripts. Our data are consistent with previous RNA-Seq studies in which these markers have been reported segregating to HC and SC cell types in mouse utricle (Burns et al., 2015; Scheffer et al., 2015).

### Hair Cell DEGs Are Enriched for Stereocilia Components, While Supporting Cell DEGs Are Enriched for Translational Machinery and Cell Adhesion

We analyzed the cell type-specific transcriptional data for functional enrichment of either biological processes or cellular components based on the gene ontology database (Supplementary Table S1). DEGs identified in the control (no heat shock) condition for both the Gfi1-Cre IP (hair cell) group (685 genes) and the GLAST-CreER IP (supporting cell) group (729 genes) were used for GO annotation analysis. Hair cell DEGs showed significant enrichment for 28 terms in the “Biological Process” category, including synapse maturation, detection of mechanical stimulus involved in the sensory perception of sound, and cilium-dependent cell motility. The enrichment for “Cellular Component” terms for the hair cell IP yielded 20 significantly overrepresented terms including GO terms for the stereocilium tip and cholinergic synapse, which in combination represent enrichment for transcripts coding for the major components of the hair cell synapse. Selected GO terms enriched in the hair cell IP are summarized in Supplementary Table S1A. The supporting cell DEGs were significantly enriched for 22 GO annotations in the “Biological Process” category, including positive regulation of acute inflammatory response, regulation of oxidative phosphorylation, and cytoplasmic translation. Supporting cell GO enrichment in the “Cellular Component” category contained terms that included both transcripts of proteins making up the lamellipodium and plasma membrane as well as enrichment for transcripts related to polysomal translational machinery. The selected GO terms in the supporting cell IP are summarized in Supplementary Table S1B.

### Validation of Cell Type Specificity in the RNA-Seq DEG Analysis

In addition to looking at canonical markers of HCs and SCs, we also validated several DEGs that were identified in our study. We selected two transcripts from the enriched DEG list for each IP (Gfi1-Cre and GLAST-CreER) and validated these using IHC. DEGs *Rbm24* and *Calb1* were each enriched in the Gfi1-Cre (hair cell) RiboTag IP (Figure 5A), and the protein products of these genes were specifically localized to HCs (Figures 5B–G). Both RBM24 and CALB1 immunoreactivity filled the entire HC body in agreement with previous literature (Golub et al., 2012). For SCs, *Rbp1* and *Tspan8* were DEGs that were enriched in the control GLAST-CreER IP compared to the Gfi1-Cre IP (Figure 5A). RBP1 immunoreactivity appeared throughout the cell body of the SCs (Figures 5I,J), and TSPAN8 immunoreactivity was localized to the SC cell membrane (Figures 5L,M). Validation of the predicted cell type specificity for these genes supports the cell-specific DEG identification of the RNA-Seq data.

### IPs From Control and Heat Shocked Utricles Reveal a Heat Shock Response in Both Hair Cells and Supporting Cells

**Figure 6:**
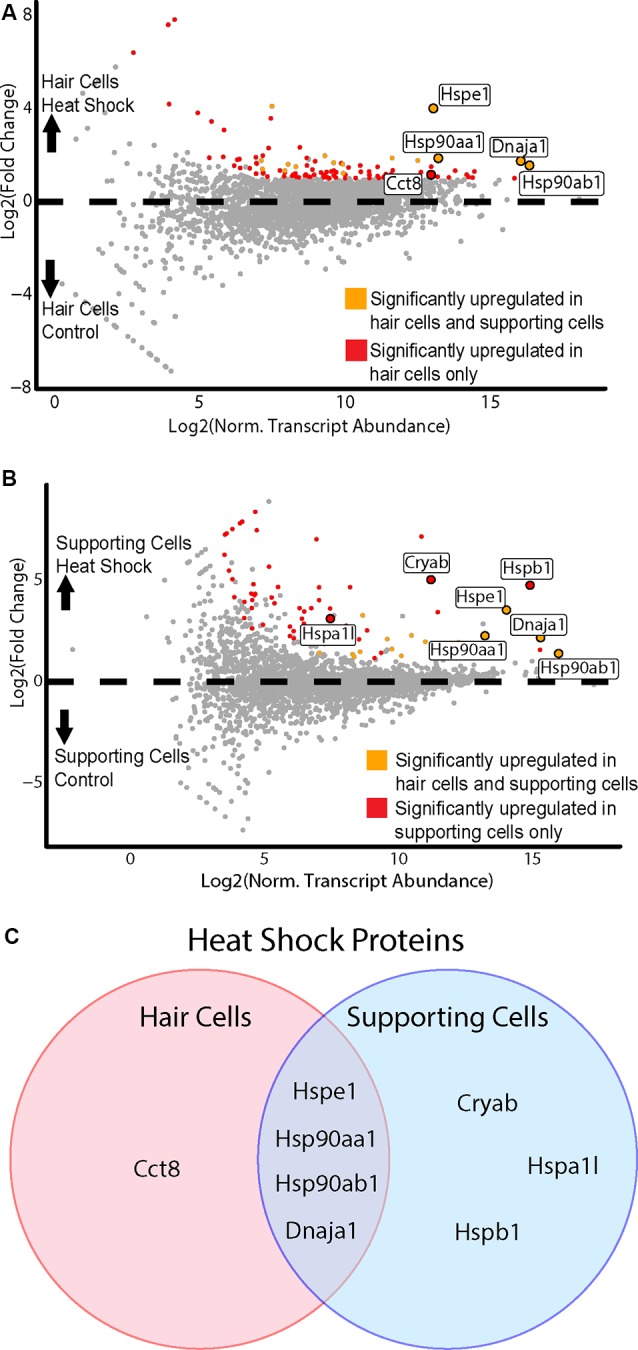
HCs and SCs demonstrate differential transcriptional responses to heat shock. **(A)** Hair cell response to heat shock. Scatterplot shows Log_2_FC values vs. normalized transcript abundance for the heat shock Gfi1-Cre IP group vs. the control Gfi1-Cre IP group. DEGs identified in both HCs and SCs are shown in orange, and DEGs identified uniquely in HCs are shown in red. Enriched HSPs are labeled and colored accordingly. **(B)** Supporting cell response to heat shock. Scatterplot shows Log_2_FC values vs. normalized transcript abundance for the heat shock GLAST-CreER IP group vs. the control GLAST-CreER IP group. DEGs identified in HCs and SCs are shown in orange, and DEGs identified uniquely in SCs are shown in red. Enriched heat shock proteins (HSPs) are labeled and colored accordingly. **(C)** Heat shock responses in HCs vs. SCs. Venn diagram illustrates the HSP transcripts significantly enriched in HCs and SCs after heat shock. Four DEGs were common between the two cell types and are members of the HSP90, HSP10, and HSP40 families. One DEG (*Cct8*) was upregulated only in HCs and is a member of the chaperonin/CCT family. Three DEGs were upregulated only in SCs (*Cryab*, *Hspa1l, Hspb1*) and are members of the HSP20, HSP70, and HSP27 families.

To examine the response to heat shock in HCs, we compared the control (no heat shock) Gfi1-Cre IP to the heat shock Gfi1-Cre IP (Figure 6A). One-hundred and eleven DEGs were identified as enriched in HCs in the heat shock condition. DEGs enriched in the heat-shocked HCs included five DEGs enriched in the GO term “unfolded protein binding” (FDR < 0.05, GO:0051082; Supplementary Table S2A). We next examined the heat shock response in SCs by comparing the control GLAST-CreER IP to the heat shock GLAST-CreER IP. Seventy DEGs were identified as enriched in the heat shock condition (Figure 6B). Six DEGs were enriched for the GO term “unfolded protein binding” (FDR < 0.05; Supplementary Table S2B). Thus, both HCs and SCs demonstrated transcriptional responses to heat shock, including induction of *Hspe1* (a member of the HSP10 family), *Dnaja1* (a member of the HSP40 family), *Hsp90aa1*, and *Hsp90ab1* (members of the HSP90 family) by both cell types. However, there were also differential responses to heat shock between HCs and SCs. *Cct8* of the chaperonin gene family was enriched only in HCs, while *Hspa1l* (HSP70 family), and *Hspb1* and *Cryab* (HSP27 and HSP20 families, respectively) were enriched only in SCs (Figure 6C). In addition to HSPs, some hair cell-specific markers, including *Calb1, Calb2*, and *Otof*, were also induced by heat shock in HCs, again suggesting a cell type-specific response to heat shock. Additional GO terms that were enriched in HCs following heat shock included “type 3 metabotropic glutamate receptor binding,” “nitric-oxide synthase regulator activity,” “calcium-dependent protein binding,” and “ion channel binding” (Supplementary Table S2). In SCs, all GO terms that were enriched after heat shock included transcripts of HSPs, though some non-HSP transcripts were enriched in SCs after heat shock. These data again support the idea that HCs have a unique and limited response to heat shock, while SCs have a more classic heat shock response.

### Immunochemistry Confirms Induction of CCT8 in HCs and HSP27 in SCs Following Heat Shock

**Figure 7:**
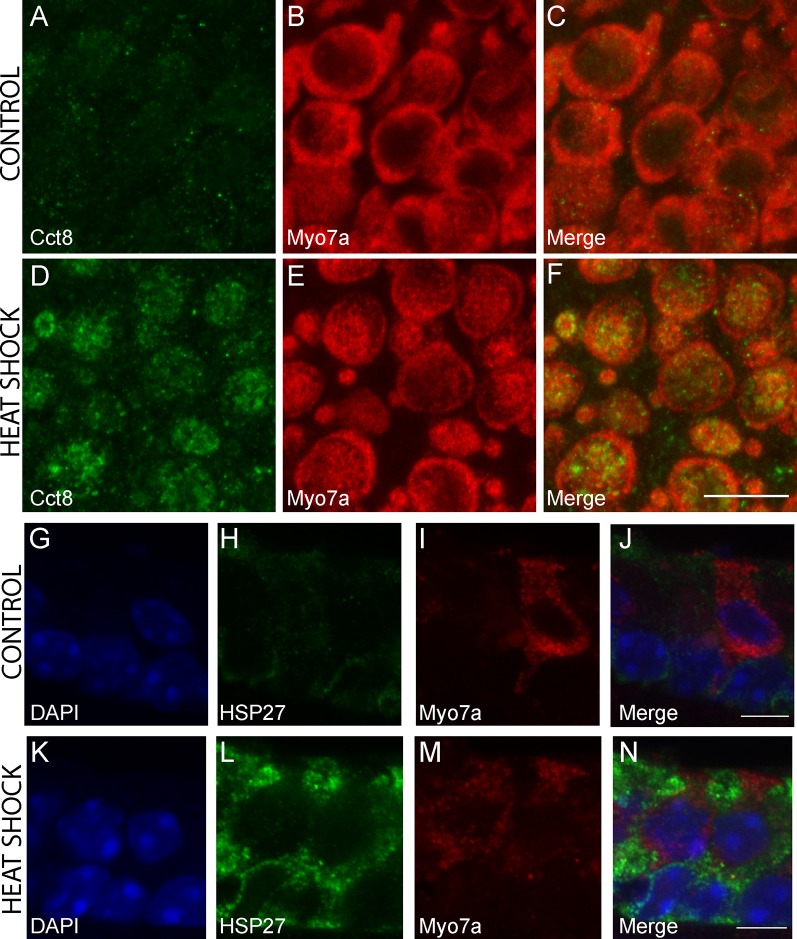
Immunohistochemical staining for cell-type-specific upregulation of HSPs after heat shock. CCT8 was the only HSP significantly upregulated in HCs but not SCs following heat shock, and HSP27 is one of three HSPs upregulated in SCs but not HCs in this study. Increased CCT8 staining (green) is observed in HCs of the heat-shocked utricle **(D–F)** as compared to the control utricle **(A–C)**. *Myo7a* was used as a hair cell marker (red; **B,E)** with merged images **(C,F)**. Images are 900 μm^2^ composites from confocal images taken at 63× magnification. Scale bar **(F)** represents 10 μm and applies to panels **(A–F)**. Increased HSP27 staining (green) is observed in SCs of the heat-shocked utricle **(K–N)** as compared to the control utricle **(G–J)**. *Myo7a* was used as a hair cell marker (red; **I,M)** and DAPI (blue) was used to visualize all nuclei **(G,K)** with merged images **(J,N)**. Images are composites taken at 63× magnification. Scale bars **(J,N)** represent 5 μm and apply to panels **(G–I)** and **(K–M)**, respectively.

The chaperonin family member *Cct8* was the only heat shock transcript that was significantly enriched in heat-shocked HCs but not in heat-shocked SCs. The *Hspb1* transcript encoding HSP27 was one of three transcripts significantly enriched in heat-shocked SCs but not in heat-shocked HCs. We examined expression of CCT8 and HSP27 in control and heat shock conditions using IHC. CCT8 immunoreactivity increased in HCs in the heat shock condition compared to the control (Figures 7A–F). CCT8 staining did not appear to be preferentially expressed in either extrastriolar or striolar regions. HSP27 immunoreactivity increased in SCs in the heat shock condition compared to the control (Figures 7G–N). These data serve as validation of the cell type-specific responses to heat shock observed in the RNA-Seq data (Figure 6C).

## Discussion

Using the RiboTag method, we isolated cell-type specific transcripts that were being actively translated by SCs and HCs of the mature mammalian utricle. Our data agree with other cell-specific RNA-Seq (FACS single-cell RNA-Seq and proteomics) isolation methods that have been utilized in the inner ear (Burns et al., 2015; Elkon et al., 2015; Scheffer et al., 2015; Tao and Segil, 2015; Hickox et al., 2017). We validated the transcriptional enrichment observed in the RiboTag IPs using IHC to show cell type-specific localization of enriched transcripts. GO analysis of the transcripts enriched in HCs in both the “Biological Process” and “Cellular Component” categories suggest that the identified transcripts are related to the hair cell functions of mechanotransduction (e.g., components of the stereocilia bundle) and neurotransmitter release (e.g., components of synaptic vesicles, synaptic membrane, and vesicle secretion). Supporting cell DEGs were enriched for a different set of both “Biological Process” and “Cellular Component” GO annotations, including categories related to translation, inflammatory response, and cellular metabolism.

We used RiboTag to examine differential responses of HCs and SCs to protective heat shock. Our data indicate that both HCs and SCs induce one or more genes associated with the HSP families HSP10, HSP40, and HSP90 in response to heat shock. HCs specifically induced CCT8 of the chaperonin family, whereas SCs specifically induced genes associated with the HSP70, HSP27, and HSP20 families after heat shock. Taken together, these data indicate that both HCs and SCs demonstrate transcriptional heat shock responses with some similarities and some cell type-specific differences. In particular, it appears SCs may have a more robust heat shock response, uniquely inducing three known HSPs associated with three different HSP families. We had previously shown that HSP70 is induced in SCs (with little induction in HCs) following heat shock using IHC (May et al., 2013). Here, we validated the induction of another HSP, HSP27, in SCs following heat shock by IHC. Compared to SCs, HCs may have a reduced heat shock response with only one heat shock family member (CCT8) detected uniquely in HCs after heat shock. We validated our finding that *Cct8* is induced in HCs following heat shock by IHC. While CCT8 was found to be specifically expressed in HCs in our study, and only after heat shock, the data regarding the expression of *Cct8* in the inner ear differ among published studies. As a group, studies which used tissue dissociation to measure cell type-specific gene expression measured high levels of *Cct8* transcripts in HCs of the auditory and vestibular systems, in both early postnatal and adult mice (Cai et al., 2015; Elkon et al., 2015; Scheffer et al., 2015; Liu et al., 2018; Ranum et al., 2019). Conversely, a study comprehensively measuring gene expression in developing and adult outer HCs using the RiboTag approach detected *Cct8* as a uniformly depleted transcript, indicating that it is not expressed in outer HCs (Chessum et al., 2018). The result of this last study, taken both with our own RiboTag results as well as our immunostaining, suggest that just as *Cct8* is induced by heat shock, it may be induced by tissue dissociation as an “acute phase” reactant. This supports the utility of the RiboTag approach in identifying cell type-specific gene expression while avoiding molecular changes induced by tissue dissociation and cell stress. CCT8 staining did not appear to be preferentially expressed in either extrastriolar or striolar regions. Without use of a type I or type II HC marker, we do not have any evidence that CCT8 expression differs between these two cell subtypes.

CCT8 is a subunit of the CCT complex, which is required for proper protein folding of both actin and tubulin (Dunn et al., 2001; Willison, 2018). In addition, CCT8 is required for assembly of the BBSome (Seo et al., 2010), a complex believed to be essential for vesicle trafficking in cilia (Klink et al., 2017). Mutations in the BBSome lead to Bardet Biedl Sydrome, a disorder caused by ciliary dysfunction. A recent study of patients with Bardet Biedl Syndrome showed abnormal DPOAEs, suggesting dysfunction of outer HCs (Esposito et al., 2017). In mammalian cells, upregulation of CCT8 specifically promotes assembly of the CCT complex without upregulation of other subunits (Noormohammadi et al., 2016). Though all CCT subunits contain heat shock elements in their promoter regions, the CCT complex has not been shown to increase following heat shock in other systems (Willison, 2018). Interestingly, ectopic expression of CCT8 in *C. elegans* led to an increase in organismal lifespan, an effect that was amplified under mild heat stress conditions (Noormohammadi et al., 2016). In summary, CCT8 is essential for actin folding and ciliary function, and our data indicate that it is uniquely induced by HCs following heat shock. Based on this finding, we suggest that HCs may be inducing HSPs that function to protect or rebuild stereocilia under heat stress conditions. These HSPs may represent potential therapeutic targets for protection of HCs under stress from noise or ototoxic drugs.

Traditionally the RiboTag method has been used to compare the enrichment of genes in the IP sample to the “input” sample (Sanz et al., 2009, 2013; De Gendt et al., 2014). The ability to directly compare IPs from different Cre lines is advantageous for studies of cell-type specific responses to stimuli. Cre models that result in incomplete or variable recombination efficiency (such as we observed with GLAST-CreER, Figure 2J) can result in missing enriched transcripts (i.e., false negative DEGs) when comparing input to IP, as low recombination efficiency can cause true signal to be covered by noise. However, a lack of comparison to the “input” can fail to properly remove noise from the analysis and result in false positive DEGs. Our approach was to directly compare IPs from different Cre lines where the genes included in the analysis were restricted to those DEGs that were significant in the IP over the “input” of the Cre driver with greater expression of that DEG (Figure 1B). This approach allowed us to directly compare IPs from different Cre lines while minimizing false positive DEGs. It is worth noting, however, that the reduced recombination efficiency in utricle SCs likely led to some supporting cell-specific transcripts not reaching the filtering requirements necessary to be included in our analysis.

The RiboTag method was employed here as a means of deriving HC and SC-specific transcriptomes, and as with any method it is worth discussing the advantages and limitations of the approach. It is important to note that in the Gfi1-Cre model, both HCs and tissue macrophages underwent Cre-mediated recombination (Matern et al., 2017), and both markers of HCs and tissue macrophages were therefore enriched in the whole-tissue Gfi1-Cre IP compared to input. Comparison of stroma-free isolated sensory epithelia IPs to whole tissue Gfi1-Cre IPs separated these markers and allowed for filtering of the macrophage-specific transcripts. Although the reliance of RiboTag on Cre lines that may undergo recombination in cell types other than the target cell type is a limitation of the RiboTag system, it may also represent a unique opportunity to discover expressed transcripts from rare and difficult-to-isolate cell populations such as tissue macrophages or other resident leukocyte-derived cells. Although this technique requires separation of the sensory epithelium from underlying stroma, it likely still results in less cellular stress than would be induced by tissue dissociation and FACS (van den Brink et al., 2017). Interestingly, even after separation of the sensory epithelium from underlying stroma, some HC markers such as *Myo7a* were not enriched in the Gfi1-Cre IP, indicating that RiboTag does not enrich for all possible cell-type specific transcripts. Similarly, two SC markers, *Gjb2* and *Gjb6*, were not enriched in the control GLAST-CreER IP, again indicating that the IP comparisons do not capture all cell-type DEGs (Figure 5A). Because the RiboTag approach results only in the collection of transcripts actively bound to ribosomes, it is possible that the transcripts associated with well-known cell-type specific proteins may not be enriched in cases where the protein is especially stable and has a low rate of turnover. Additionally, transcripts of proteins with longer half-lives may not be actively translated at all timepoints. One limitation of this study is that it captures the translatome response to heat shock in the utricle at a single timepoint, 2 h post-heat shock. This timepoint corresponds to the peak of *HSP70* mRNA expression in heat shocked utricles (Cunningham and Brandon, 2006), but there may be subtleties of the cell-type specific responses to heat shock that appear at other timepoints in the stress response. It is also worth noting that the limited efficiency of the IP, which is estimated to capture roughly 25% of the HA-tagged ribosomes from tissue lysates (Sanz et al., 2009), may prevent the enrichment of some well-known cell-type specific transcripts.

This study used the RiboTag method to obtain cell type-specific transcripts from inner ear tissues. We first characterized the specificity of the Cre mouse models in a tdTomato reporter line. We isolated transcripts from SCs and HCs and validated the methodology by showing RNA-Seq enrichment of known cell type-specific markers and IHC of novel markers. The Gene Ontology enrichment for each cell type suggests that SCs may be functionally enriched in transcripts related to translational machinery and inflammatory responses, while HCs are enriched predominately for structural transcripts related to stereocilia and mechanotransduction. We have confirmed that the Gfi1-Cre model also enriches for the tissue macrophages of the inner ear (Matern et al., 2017), and these transcripts can be revealed by comparison to IP of isolated stroma-free utricle sensory epithelium. Finally, we studied the effect of heat stress on cultured utricles and found that the heat shock response is present at the transcriptional level in both HCs and SCs, but there are differences between the responses of the two cell types. Our data suggest that induction of *Hspa1l*, *Hspb1*, and *Cryab*, associated with the HSP70, HSP27, and HSP20 families, respectively, was restricted to SCs following heat shock, while HCs show unique induction of CCT8 of the chaperonin family. One interpretation of these data is that the hair cell response to heat stress is limited and focused on maintenance of stereocilia, while SCs display a more robust and classical heat shock response. Additional transcripts that are induced by heat shock but do not encode classical HSPs (*Snap25*, *Calm1*, *Calm2*, *Calm3*, *Hnrnpm* in HCs; *Rpph1*, *Arl5c*, *Edn1* in SCs) may provide insight into the full range of responses in both HCs and SCs. The proteins encoded by these additional transcripts may modulate the protection provided by classical HSPs and may therefore represent additional critical elements of the cell type-specific responses to heat shock.

## Data Availability Statement

The datasets generated and analyzed for this study can be found in the GEO (GSE139593) and gEAR (https://umgear.org/p?s=0cde9c63) databases. Raw counts files and differential expression results are also available as part of the Supplementary Material.

## Ethics Statement

The animal study was reviewed and approved by National Institute on Deafness and Other Communication Disorders.

## Author Contributions

ES, MR, LM, and NW performed experiments. ES, MR, and LC wrote the manuscript. DM, EB, and RM prepared libraries, performed RNA sequencing and provided bioinformatic guidance. RH and LC conceived of the project, guided data analyses, and critiqued the manuscript.

## Conflict of Interest

The authors declare that the research was conducted in the absence of any commercial or financial relationships that could be construed as a potential conflict of interest.

## Footnotes

^1^https://umgear.org/p?s=0cde9c63
