# Supplementary material for: Cell-Specific Transcriptional Responses to Heat Shock in the Mouse Utricle Epithelium
Source: Front Cell Neurosci. 2020 May 15;14:123. doi: 10.3389/fncel.2020.00123 (PMC7247426; doi:10.3389/fncel.2020.00123)
Supplement: Supplementary file 6 [file Data_Sheet_1.docx]

Supplementary Material

# Supplementary Data Legends

## Supplementary Table 1

**Supplementary Table 1.** Selected GO terms for Gfi1-Cre and GLAST-CreER IP enrichment comparisons. **A**) Sample of significantly overrepresented ‘Biological Process’ and ‘Cellular Component’ GO terms returned using PANTHER on the 685 DEGs selected from the Gfi1-Cre IP (HC) enrichment compared to GLAST-CreER IP (SC). The name of the GO term and its accession number, the number of genes in each GO term, the fold of overrepresentation of DEGs in that GO term from the PANTHER query, and the FDR are shown in each column. **B**) Sample of significantly overrepresented ‘Biological Process’ and ‘Cellular Component’ Go terms returned using PANTHER on the 729 DEGs selected from the GLAST-CreER IP enrichment compared to Gfi1-Cre IP.

## Supplementary Table 2

**Supplementary Table 2.** Selected GO terms for Gfi1-Cre and GLAST-CreER IP enrichment comparisons post-heat shock. **A**) Sample of significantly overrepresented ‘Molecular Function’ GO terms returned using PANTHER on the 111 DEGs selected from the Gfi1-Cre IP (HC) enrichment post-heat shock compared to Gfi1-Cre IP without heat shock. The name of the GO term and its accession number, the number of genes in each GO term, the fold of overrepresentation of DEGs in that GO term from the PANTHER query, and the FDR are shown in each column. **B**) Sample of significantly overrepresented ‘Biological Process’ and ‘Cellular Component’ Go terms returned using PANTHER on the 70 DEGs selected using the stringent cutoff criteria from the GLAST-CreER IP (SC) enrichment post-heat shock compared to GLAST-CreER IP without heat shock.

## Supplementary Table 3

**Supplementary Table 3.** The full raw count file used for differential expression analyses in this study.

## Supplementary Table 4

**Supplementary Table 4.** Results of the differential expression comparison between input and immunoprecipitate (IP) for each experimental condition. Experimental conditions include Gfi1-Cre in stroma-free isolated sensory epithelium (sfHC) in control (co) and in heat shock (hs), Gfi1-Cre in whole-tissue utricle (wtHC) in co and in hs, and GLAST-CreER in whole tissue utricle (SC) in co and in hs.

## Supplementary Table 5

**Supplementary Tables 5.** Results of the differential expression comparisons between sfHC-co and wtHC-co (**A**), sfHC-co and SC-co (**B**), sfHC-co and sfHC-hs (**C**), SC-co and SC-hs (**D**), and wtHC-co and wtHC-hs (**E**). Each results sheet includes two additional tabs listing only the genes found to be expressed significantly more in each of the conditions.
